# Local torsion of distal femur is a risk factor for patellar dislocation

**DOI:** 10.1186/s13018-023-03646-3

**Published:** 2023-03-03

**Authors:** Chongyi Fan, Yingzhen Niu, Fei Wang

**Affiliations:** Department of Joint Surgery, Hebei Medical University Affiliated Third Hospital, Shijiazhuang, 050051 Hebei China

## Abstract

**Purpose:**

It has been widely reported that femoral anteversion is a risk factor for patellar dislocation. This study aims to evaluate whether internal torsion of the distal femur is noticeable in patients without increased femoral anteversion and to assess whether it is a risk factor for patellar dislocation.

**Methods:**

A retrospective analysis was conducted on 35 patients (24 females, 11 males) with recurrent patellar dislocation but without increased femoral anteversion treated in our hospital from January 2019 to August 2020. All patients underwent knee X-rays, digital radiography of lower-limbs, and CT scans of hip, knee, and ankle joints to measure femoral anteversion angle, distal femoral torsion angle, TT–TG and Caton-Deschamps index. Thirty-five control cases were matched on age and sex to compare the difference of anatomic parameters between the two groups, and the logistic analysis was used to analyze risk factors for patellar dislocation. Perman correlation coefficient was used to evaluate the correlation among femoral anteversion, distal femoral torsion and TT–TG.

**Results:**

Greater distal femoral torsion was still observed in patients with patellar dislocation but without increased femoral anteversion. The torsion angle of distal femur, TT–TG distance and incidence of Patella Alta in patients with patellar dislocation were greater than those in control group, and the inter-group differences were statistically significant (*P* < 0.05). The torsion angle of distal femur (OR = 2.848, *P* < 0.001), TT–TG distance (OR = 1.163, *P* = 0.021) and Patella Alta (OR = 3.545, *P* = 0.034) were risk factors for patellar dislocation. However, no significant correlation was found among femoral anteversion, distal femoral torsion and TT–TG in patients with patellar dislocation.

**Conclusion:**

On the condition that femoral anteversion did not increase, increased distal femoral torsion was commonly observed in patients with patellar dislocation, which represents an independent risk factor for patellar dislocation.

**Supplementary Information:**

The online version contains supplementary material available at 10.1186/s13018-023-03646-3.

## Introduction

It has been widely reported that femoral anteversion is a risk factor for patellar dislocation [1–5]. The femoral anteversion is defined as the angle between the line going through the femoral head and the femoral neck and the tangential line of posterior femoral condyle, indicating the overall femoral torsion [6]. To determine the ideal segment for performing femoral osteotomy, many authors have recently evaluated the position of the increased femoral anteversion. It has been found that the increased of the femoral shaft and distal femoral torsion is most noticeable in patients with patellar dislocation [1, 7–9]. However, many authors previously found that patients with patellar dislocation often developed bone malformations of the femoral condyle [10–13], and therefore, bone malformations of the distal femur is likely to result in internal torsion of the distal femur.

The femoral anteversion evaluates the overall torsion of the femur and does not reflect the location of the torsion. The internal and external torsion between different segments of the femur can cancel each other out, keeping the femoral torsion within the normal range. The torsion of the distal femur causes the knee joint to become the "Inward-pointing knee," and the Q Angle increases, increasing the lateral sagittal tension of the knee joint [14]. As the times of ossification centers of femoral shaft and femoral condyles are different, the development of the femoral condyle is earlier than that of the femoral shaft [15]. Previously, all authors based their evaluation of the femoral torsion on the femoral anteversion, which could not properly reflect the local torsion of femoral condyle. For patients with patellar dislocation but without significant increase in femoral anteversion, it is still unknown whether the abnormal bony structure only appears in the distal end of the femur. Meanwhile, dysplasia of the femoral condyle is likely to be accompanied by abnormal development of soft tissues such as muscles and tendons attached to the femoral condyle.

Derotational femoral osteotomy has been confirmed reliable in correcting poor rotatory lines of the lower extremity in patients with increased femoral anteversion. However, no consensus has been reached on the threshold of correction of femoral anteversion. Several scholars have reported that torsional deformity of femoral shaft plays a major role in the femoral torsion, while torsion of distal femur plays a minor role [1, 7, 9]. It has not yet been reported whether local torsion of distal femur is an independent risk factor for patellar dislocation in patients with patellar dislocation but free of significant femoral anteversion. Thus, this study aims to compare distal femoral torsion in patients with patellar dislocation (without increased femoral anteversion) with that in patients without patellar dislocation and to explore whether distal femoral torsion is an independent risk factor for patellar dislocation.

## Materials and methods

*Inclusion criteri*a for patients with patellar dislocation:

1. History of patellar dislocation (at least 3 times),
2. Closure of epiphysis confirmed by preoperative CT scans,
3. Preoperative lateral radiographs of knee joint and CT scan of hip, knee and ankle
4. Range of normal femoral anteversion: 9°–17°. (It is generally accepted that the normal range of femoral anteversion is between 10°and 15°. Due to possible measurement differences, we prioritized measurement on the control group and found that the femoral anteversion ranged from 9° to 17°).

### Exclusion criteria

1. History of other knee surgeries
2. Developmental hip dysplasia
3. History of fracture of femur or tibia on the affected side.

A total of 89 patients with patellar dislocation were treated in our hospital from January 2019 to August 2020. Based on the above inclusion/exclusion criteria, thirty-five patients (24 females and 11 males) were included in this study. The control group consisted of patients with no radiological signs of trochlear dysplasia and no clinical signs of subjective/objective patellar dislocation. The control group was matched on age and sex, with a total of 35 patients included. Refer to Table 1 for patient demographics. This study was approved by the Ethics Committee of our hospital and informed consent was obtained from all participants.

**Table 1:** Patient demographics

Group	Patient group(*n* = 35)	Control group(*n* = 35)	*P*
Mean age, yrs (SD)	21.08(5.59)	20.34(6.07)	n.s
Female:male, n	24:11	25:10	n.s
Mean BMI, kg/m^2^ (SD)	24.09(4.52)	23.34(4.31)	n.s
Side,R:L	16:19	18:17	n.s

Dejour type	
A	22
B	5
C	4
D	4

### Imaging measurement

#### CT evaluation of femoral torsion

**Fig. 1:**
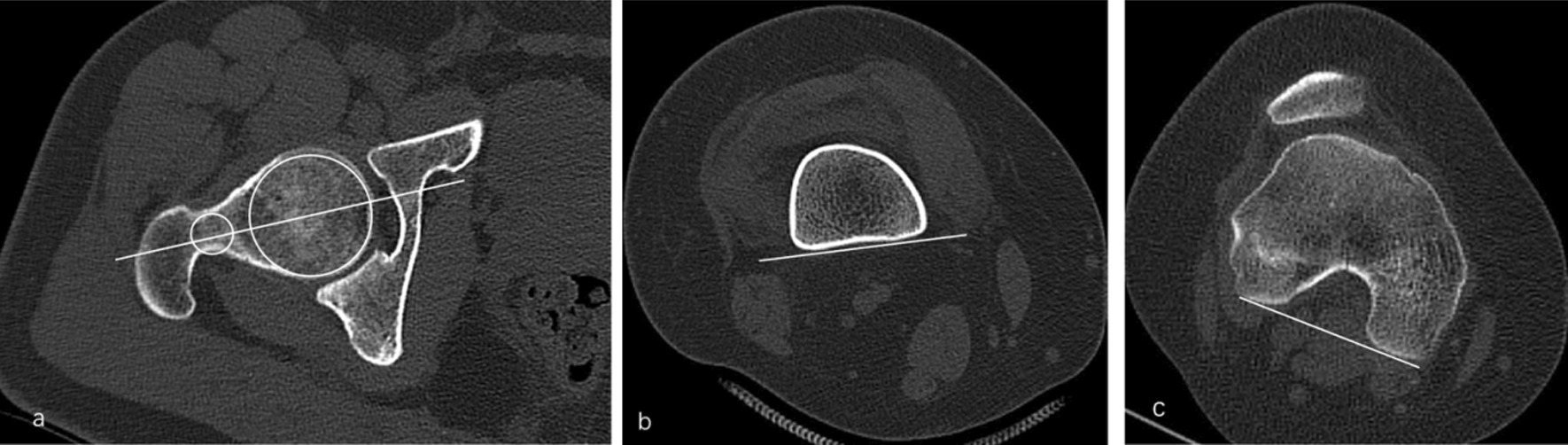
Three lines were drawn to evaluate the femoral torsion in CT images. The first line passes through the center of the femoral head and neck. (FNA,** a**). The second line is the tangent line to the gastrocnemius muscle in the posterior compartment of the leg (DFL,** b**). The third line is the tangent line to the posterior condyles (PCL,** c**). The angle between FNA and PCL (femoral anteversion)and the angle between DFL and PCL (distal femoral torsion) were measured.

Three lines were drawn to evaluate the femoral torsion in CT images. The first line passes through the center of the femoral head and neck (FNA, Fig. 1a). The second line is the tangent line to the gastrocnemius muscle in the posterior compartment of the leg (DFL, Fig. 1b) and the third line is the tangent line to the posterior condyles (PCL, Fig. 1c). The angle between FNA and PCL (femoral anteversion angle, FAA) and the angle between DFL and PCL (distal femoral torsion, DFA) were measured.

### TT–TG measurement

Superimpose axial images of:

- Proximal aspect of femoral trochlea.
- Proximal aspect of tibial tuberosity (TT).

**Fig. 2:**
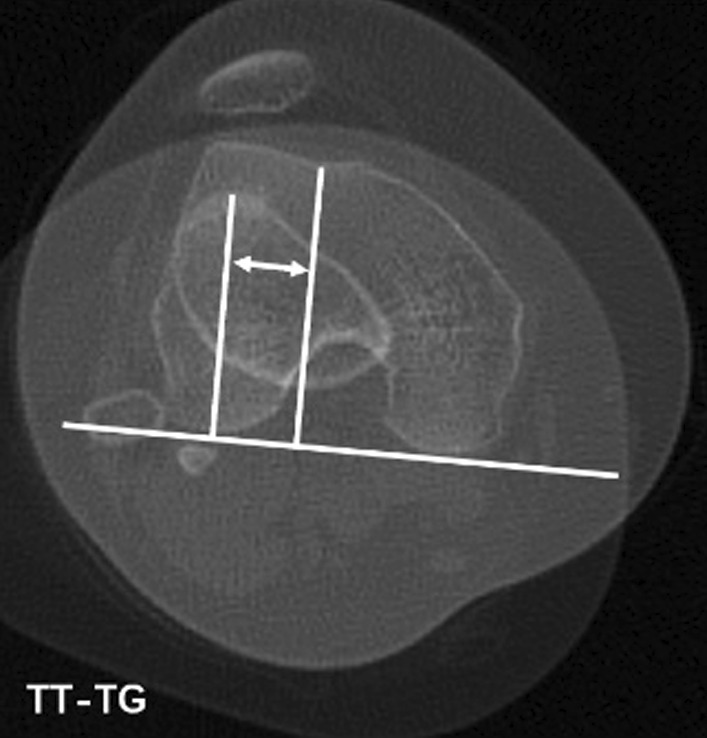
Draw a line along the posterior femoral condyles, and then draw the following lines perpendicular to this line. Bisecting the tibial tuberosity (TT). Bisecting the trochlear groove sulcus (TG). Measure the distance between TT and TG = TT–TG distance

Draw a line along the posterior femoral condyles, and then draw the following lines perpendicular to this line:

- Bisecting the tibial tuberosity.
- Bisecting the trochlear groove sulcus (TG).
- Measure the distance between TT and TG = TT–TG distance (Fig. 2).

*Patellar height* The Caton-Deschamps index was used to measure patellar height.

A: distance between the anterior angle of the tibial plateau, to the most inferior aspect of the patellar articular surface.

B: patellar articular surface length.

Caton-Deschamps index = A/B. A ratio more than 1.2 was defined as patella alta.

### Statistical analysis

Statistical analysis software of SPSS 21.0 (SPSS, USA) was used for data analysis. The parameters were measured by two orthopedists and the interclass correlation coefficient (ICC) was calculated to determine the reliability of each parameter, with a value greater than 0.75 indicating excellent agreement. Among the four anatomical parameters, three (FAA, DFA and TT–TG distance) were continuous variables while the Patella Alta was the categorical variable. Monofactor analysis was first conducted. Chi-square test was used to compare categorical variables between groups; independent T-Test was used to compare continuous variables between groups when the homogeneity of variance and normal distribution was satisfied. Otherwise, Mann–Whitney U Test would be used for test. Then Binary Logistic regression analysis was used to analyze the correlation between anatomical parameters and patellar dislocation. An alpha of 0.05 was used on both sides. The Pearson correlation coefficient was also used to analyze the correlation among the FAA, DFA and TT–TG. A *P* value less than 0.05 was considered statistically significant. All values were expressed as mean ± standard deviation.

## Results

All parameter results are shown in Tables 2, 3, 5. The interobserver correlation coefficient of each anatomic measurement showed excellent agreement (> 0.80) (Additional file 1: Table).

**Table 2:** Comparison of anatomic parameters between patient group and control group

Group	Patient group(*n* = 35)	Control group(*n* = 35)	*P*
FAA,°	13.11 (2.68)	12.14 (2.63)	0.131
DFL-PCL,°	10.60 (2.25)	6.85 (1.51)	< 0.001
TT–TG,mm	15.05 (3.95)	12.70 (4.04)	0.016
Patella alta, yes/no,n	13/22	5/30	0.029

FAA: femoral anteversion angle DFL-PCL: distal femoral torsion TT–TG: the distance between tibial tuberosity and trochlear groove sulcus

**Table 3:** The results of binary logistic regression analysis of patellar dislocation

Parameter	*β*	OR	95% CI	*P*
DFL-PCL	1.047	2.848	1.744, 4.651	< 0.001
TT–TG	0.151	1.163	1.023, 1.323	0.021
Patella alta	1.266	3.545	1.102, 11.411	0.034

*DFL-PCL* distal femoral torsion; *TT–TG* the distance between tibial tuberosity and trochlear groove sulcus

This study revealed greater distal femoral torsion was still noticeable in patients with patellar dislocation but without increased femoral anteversion. The torsion angle of distal femur, TT–TG distance and incidence of Patella Alta in patients with patellar dislocation were greater than those in control group, and the inter-group differences were statistically significant (*P* < 0.05) (Table 2).

Three variables (the torsion angle of distal femur, TT–TG distance and Patella Alta) that showed statistically significant differences in monofactor analysis were included in Logistic regression analysis. The results found that increased femoral anteversion, increased TT–TG distance and Patella Alta were risk factors for patellar dislocation (Table 3).

Pearson correlation analysis revealed significant correlation among all participants’FAA, DFA and TT–TG distance. (Table 4). However, subgroup analysis of patients with patellar dislocation found significant correlation between FAA and DFA (*r* = 0.649, *P* < 0.001), but neither of them had significant correlation with TT–TG distance (Table 5).

**Table 4:** Pearson correlation coefficients for all participant parameters

Parameter	*r*-value	
	TT–TG	DFL-PCL
FAA	**0.254**	**0.573**
DFL-PCL	**0.335**	–

Bolded values indicate statistical significance (*P* < 0 .05)

*FAA* femoral anteversion angle; *DFL-PCL* distal femoral torsion; *TT–TG* the distance between tibial tuberosity and trochlear groove sulcus

**Table 5:** Pearson correlation coefficient of patient group parameters

Parameter	*r*-value	
	TT–TG	DFL-PCL
FAA	0.075	**0.649**
DFL-PCL	0.117	–

Bolded values indicate statistical significance (*P* < 0 .05)

*FAA* femoral anteversion angle; *DFL-PCL* distal femoral torsion; *TT–TG* the distance between tibial tuberosity and trochlear groove sulcus

## Discussion

**Fig. 3:**
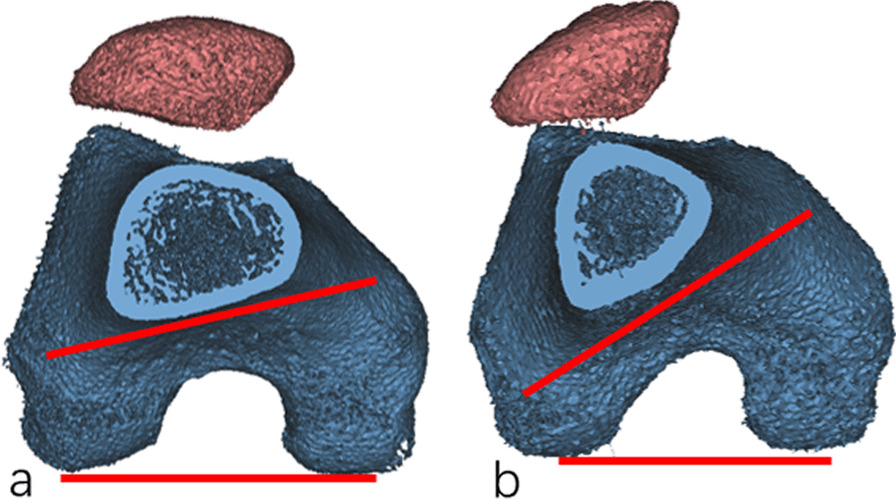
**a** distal femoral torsion in patients without patellar dislocation. **b** distal femoral torsion in patients with patellar dislocation. By comparison, a more noticeable torsion of distal femur can be observed in patients with patellar dislocation

This study revealed that patients with patellar dislocation generally showed higher distal femoral torsion, even when the femoral anteversion did not increase. The femoral anteversion has been widely used to evaluate the femur torsion in the lower extremity, which extends from the hip joint to the knee joint. As this span is very large, the local torsion of the femur cannot be adequately reflected. Previously, many authors observed morphological changes in posterior condyle and change of torsion angle of posterior femoral condyle in patients with patellar dislocation, and they generally believed that the increase in posterior medial condyle and shortening of posterior lateral condyle together contributed to internal torsion of distal femur [10, 11]. The torsion of the posterior femoral condyle (PCA) was measured by drawing a line passing through the transepicondylar axis and the posterior condyle. However, since the femoral epicondylar axis was part of the femoral condyle, this measurement could not reflect the overall abnormal torsion of the femoral condyle. By comparing the distal femoral torsion, we found that, not only the morphology of the posterior femoral condyle changed, but the axial torsion of the overall femoral condyle occurred (Fig. 3).

A mechanical evaluation of femoral torsion conducted by Kaiser et al. found that a FAA more than 20° could significantly increase the lateral sagittal tension [4]. By using three-dimensional finite element method, Liska et al. [16] analyzed the change of pressure on patellofemoral joint caused by the increased femoral anteversion. Ferràs-Tarragó et al. [17] conducted the three-dimensional finite element analysis and found that final femoral anteversion would not be affected as long as the osteotomy plane was perpendicular to the femoral shaft. However, instead of considering possible malformation of the femoral condyle in patients with patellar dislocation, all these authors only increased the angle of the overall femoral torsion by simulations. In patients with patellar dislocation, morphological dysplasia of the anterior femoral condyle is quite common, such as reduced trochlear depth, trochlear dysplasia and proximal femoral bumps and there are also obvious deformities of the posterior femoral condyle [18]. Roger et al. [10] reported the shortening of posterior femoral condyle in patients with patellar dislocation, but failed to reveal the correlation between torsion angle of posterior femoral condyle and patellar dislocation. Liu et al. [11] also reported abnormal morphology of posterior femoral condyle in patients with patellar dislocation, enlarged posterior medial condyle and shortened posterior lateral condyle. Gillespie D et al. believed that it is the overall multi-plane dysplasia of the lateral condyle rather than the shortening of the posterolateral condyle of femur only that contributes to the patellar instability [19]. Our study confirmed that patients with patellar dislocation showed more noticeable internal rotation of distal femur, which indicates the torsion of femoral condyle is the overall axial torsion, and that the overall abnormal development of femoral condyle may be the key factor for patellar dislocation.

Many factors contribute to the patellar dislocation. Besides the abnormal bone structure, abnormal soft tissue is also a key factor for the patellar dislocation. In an abnormal femoral condyle, muscles and tendons attached to the bone all undergo different degrees of soft tissue dysplasia. To some extent, musculoskeletal integration caused by patellar dislocation is an important pathogenic mechanism of patellar instability. Yuko Suzuki et al. discovered that dysplasia of femoral condyle is likely to go with abnormal development of muscles and tendons attached to the femoral condyle [15]. Dong et al. have reported quadriceps atrophy in patients with patellar dislocation [20], and compression of the lateral retinaculum is also a leading risk factor for patellar dislocation. Together with high compression of lateral retinaculum, reduction in contractility of the medial muscles of the knee joint caused by inward axial torsion of the femoral condyle result in the patellar dislocation.

The natural knee kinematics has been extensively studied and the femoral epicondylar axis is widely accepted as the flexion axis of knee joint [21] and meanwhile the direction and position of femoral condyle are significantly related to the knee kinematics [22]. Our study discovered even if the femoral anteversion was within the acceptable range, the femoral epicondylar axis in patients with patellar dislocation was still in a more internal position. In the extended position, excessive internal rotation of the femur increased the vector force on the lateral side of the patella, while during flexion, changes of the posterior femoral condyle, especially the shortening of the posterior lateral condyle of the femur would lead to increased external rotation, eventually resulting in flexion instability. This suggests that the surgeon should notice the abnormal knee kinematics when treating patients with patellar dislocation, and it is not just the kinematics of patellofemoral joint, but also the tibiofemoral joint.

Dejour et al. [23] found the FAA in patients with patellar dislocation was 15.6°and 10°in healthy controls. It has been confirmed that increased femoral anteversion is widely observed in patients with patellar dislocation. Ignoring the local torsion of the femoral condyle in patients with patellar dislocation, all previous authors used the femoral anteversion to evaluate the internal rotation of the distal femur. Zhang, Imhoff FB, Nelitz M, et al. have all reported good surgical effects of derotational distal femoral osteotomy on patients with patellar dislocation accompanied with increased femoral anteversion [24–26], but surgeons have failed to reach a consensus on the threshold of femoral anteversion [30]. It is generally accepted that FAA more than 25°or 30° is an indication for this surgical procedure. However, for patients with FAA less than 20°, as reported by Dejour et al. there is still an increase in femoral anteversion. Source of this part of lower-limb torsion needs to be explored. Our study confirmed distal femoral torsion was dominant in these patients, and that malformed development of the femoral condyle resulted in the axial torsion of the femoral condyle. However, the fact is that neither supracondylar osteotomy nor subtrochanteric osteotomy can change the abnormal torsion of the femoral condyle caused by abnormal development of the femoral condyle. Therefore, surgeons must be very careful when applying osteotomies to solve the problem of distal femoral torsion. Moreover, trochlear dysplasia can be found in almost all patients with recurrent patellar dislocation. In our study, 22 patients underwent low-grade trochlear dysplasia (Dejour A) and 13 patients developed high-grade trochlear dysplasia (Dejour B, C, D). We believe the development of anterior femoral condyle and posterior femoral condyle correlates with each other, and that it is the overall abnormality of femoral condyle that leads to patellar instability. Recurrent patellar dislocation may also contribute to cartilage damage [29], and if we do not solve the problems resulting from bone abnormalities, patients may develop patellofemoral arthritis earlier.

It has been widely reported that TT–TG is a risk factor for patellar dislocation. Many factors affect TT–TG. Torsion of the femur is one of them. Previously, Yang et al. reported that TT–TG decreased after derotational osteotomy [27], and Imhoff also reported a significant correlation between femoral torsion and TT–TG [28]. Results of our study showed, compared to normal people, in patients with patellar dislocation, the femoral anteversion and torsion of femoral condyles would result in the increase in TT–TG. In this study, no correlation was found between TT–TG and distal femoral torsion in patients with patellar dislocation. It indicates that local torsion of distal femur widely exists in patients with patellar dislocation and exerts little effect on TT–TG and that high variability of TT–TG is more likely to result from the increased femoral anteversion, tibial torsion and lateralization of tibial tubercles.

There are some limitations to our study. First, the sample size of our study is relatively small. A larger sample size is needed to evaluate the ratio of each femoral anteversion so as to better consider indications for femoral torsion. Second, femoral anteversion, femoral condylar torsion, TT–TG and trochlear dysplasia may be correlated with each other, and pathological factors for femoral condylar abnormalities need to be further explored. Third, there is still no clinical evidence on how to treat the distal femoral torsion and whether surgical treatment should be used to correct the morphology of femoral condyles.

## Conclusion

On the condition that femoral anteversion did not increase, increased distal femoral torsion was noticeable in patients with patellar dislocation, which represents an independent risk factor for patellar dislocation. This reminds the surgeons to beware of the overall dysplasia of the femoral condyle, especially the abnormal femoral epicondylar axis when treating patients with patellar dislocation.

## Supplementary Information

**Additional file 1**. Inter- and Intraobserver Reliability of the Different Measurements.

## Data Availability

The detailed data and materials of this study are available from the corresponding author via e-mail on reasonable request.
