# Supplementary material for: Local torsion of distal femur is a risk factor for patellar dislocation
Source: J Orthop Surg Res. 2023 Mar 3;18:163. doi: 10.1186/s13018-023-03646-3 (PMC9983249; doi:10.1186/s13018-023-03646-3)
Supplement: Supplementary file 1 — Additional file 1. Inter- and Intraobserver Reliability of the Different Measurements. [file 13018_2023_3646_MOESM1_ESM.docx]

Supplemental Table

Inter- and Intraobserver Reliability of the Different Measurements

|  | Intraclass Correlation Coefficient (95% CI) | |
| --- | --- | --- |
| Parameter | Interobserver ICC (95% CI) | Intraobserver ICC (95% CI) |
| FAA | 0.810（0.710-0.877） | 0.895（0.831-0.935） |
| DFL-PCL | 0.842（0.757-0.898） | 0.914（0.862-0.947） |
| TT-TG | 0.970（0.953-0.982） | 0.985（0.976-0.991） |
| CDI | 0.962（0.940-0.976） | 0.981（0.969-0.988） |
